# Imaging glymphatic response to glioblastoma

**DOI:** 10.1186/s40644-023-00628-w

**Published:** 2023-10-30

**Authors:** Jasleen Kaur, Guangliang Ding, Li Zhang, Yong Lu, Hao Luo, Lian Li, Edward Boyd, Qingjiang Li, Min Wei, Zhenggang Zhang, Michael Chopp, Quan Jiang

**Affiliations:** 1 Department of Neurology, Henry Ford Health System, Detroit, MI USA; 2 Department of Physics, Oakland University, Rochester, MI USA; 3 Department of Radiology, Michigan State University, Lasing, MI USA; 4 Department of Physiology, Michigan State University, Lasing, MI USA; 5 Department of Neurology, Wayne State University, Detroit, MI USA

**Keywords:** Glymphatic system, Glioblastoma, MRI, Kinetic modeling, Tumor cell infiltration, Drug delivery

## Abstract

**Background:**

The glymphatic system actively exchanges cerebrospinal fluid (CSF) and interstitial fluid (ISF) to eliminate toxic interstitial waste solutes from the brain parenchyma. Impairment of the glymphatic system has been linked to several neurological conditions. Glioblastoma, also known as Glioblastoma multiforme (GBM) is a highly aggressive form of malignant brain cancer within the glioma category. However, the impact of GBM on the functioning of the glymphatic system has not been investigated. Using dynamic contrast-enhanced magnetic resonance imaging (CE-MRI) and advanced kinetic modeling, we examined the changes in the glymphatic system in rats with GBM.

**Methods:**

Dynamic 3D contrast-enhanced T1-weighted imaging (T1WI) with intra-cisterna magna (ICM) infusion of paramagnetic Gd-DTPA contrast agent was used for MRI glymphatic measurements in both GBM-induced and control rats. Glymphatic flow in the whole brain and the olfactory bulb was analyzed using model-derived parameters of arrival time, infusion rate, clearance rate, and residual that describe the dynamics of CSF tracer over time.

**Results:**

3D dynamic T1WI data identified reduced glymphatic influx and clearance, indicating an impaired glymphatic system due to GBM. Kinetic modeling and quantitative analyses consistently indicated significantly reduced infusion rate, clearance rate, and increased residual of CSF tracer in GBM rats compared to control rats, suggesting restricted glymphatic flow in the brain with GBM. In addition, our results identified compromised perineural pathway along the optic nerves in GBM rats.

**Conclusions:**

Our study demonstrates the presence of GBM-impaired glymphatic response in the rat brain and impaired perineural pathway along the optic nerves. Reduced glymphatic waste clearance may lead to the accumulation of toxic waste solutes and pro-inflammatory signaling molecules which may affect the progression of the GBM.

## Background

Glioblastoma multiforme (GBM) is a type of glioma that arises from astrocytes and is the most aggressive form of brain tumor, classified as grade 4 in malignancy by the World Health Organization [1]. In the United States, roughly 14,000 new instances of GBM are diagnosed each year. The average survival rate for people with GBM is about 14–16 months after diagnosis. Treatments after diagnosis include surgery followed by radiation therapy and chemotherapy with temozolomide [2]. With this form of tumor, fewer than 5% of patients survive for five years or more [3]. The microenvironment in GBM facilitates the invasion of tumor cells into the surrounding healthy brain tissue [4].

The glymphatic system, discovered in 2012, provides a pathway for cerebrospinal fluid (CSF) to enter from the subarachnoid space into the brain parenchyma to interact with the interstitial fluid (ISF) and interstitial waste solutes, using the perivascular spaces surrounding the blood vessels, and is facilitated by aquaporin-4 (AQP4) water channel proteins present at astrocyte end-feet. CSF-ISF mixture along with the interstitial waste solutes leave the brain parenchyma via the perivenous spaces to re-enter the subarachnoid space [5–7]. Waste solutes may then exit the cranium through newly discovered meningeal lymphatic vessels, perineural spaces along cranial nerves and spinal nerves to finally reach the deep cervical lymph nodes [8–11], or they may drain into the blood via the arachnoid granulations and specific transport mechanisms present at the blood-brain barrier (BBB) [6, 9, 10, 12, 13].

The glymphatic system aids in clearing the brain of amyloid-beta (Aβ), a protein waste that is a characteristic of Alzheimer’s disease (AD) [6, 14, 15]. Dysfunction of the glymphatic system and reduced waste clearance from the brain have been reported in various neurological disorders including AD [16–18], small vessel disease (SVD) [19, 20], traumatic brain injury (TBI) [21–27], stroke [28–30], diabetes [31], migraine [32], microinfarcts [33, 34], glaucoma [35], etc.

Recently, the dorsal meningeal lymphatic vessels have been demonstrated to be crucial for producing an effective immune response against brain tumors and draining glioma cells into cervical lymph nodes (CLNs), thereby playing a role in extracranial metastasis [36]. Another excellent study demonstrated that the basal meningeal lymphatic vessels serve as a major route as compared to dorsal meningeal lymphatic vessels in draining CSF macromolecules to the extracranial lymphatics and that aging impairs the meningeal lymphatic vessels and CSF drainage [37]. Since the meningeal lymphatic vessels are the potential efflux pathway for the glymphatic system and further drain the interstitial waste solutes to the CLNs [38–41], we speculate that within the brain parenchyma, the glymphatic pathway may facilitate tumor invasion by providing a perivascular route for GBM cells to migrate away from the primary tumor location and invade healthy brain tissue. The highly invasive nature of GBM cells is a primary reason for the failure of conventional treatment methods [42]. Exploring tumor cell infiltration techniques and devising anti-invasive therapies are thus of great importance. Since the glymphatic system allows CSF to circulate within the brain parenchyma, it may also facilitate efficient drug delivery for GBM via intra-cisterna magna (ICM) injection. Thus, a better understanding of the functioning of the glymphatic system with GBM may have important implications for both the progression and treatment of GBM.

Using dynamic contrast-enhanced magnetic resonance imaging (CE-MRI) and our kinetic modeling, the aim of the present study is to investigate the response of the glymphatic system in rats to GBM. Since within the brain parenchyma, the olfactory bulb is sensitive to the changes in the glymphatic system influx and efflux [5, 43], we assessed the olfactory bulb and the whole brain for changes in the glymphatic function in response to GBM. In addition to the glymphatic system response to GBM, we also examined the CSF tracer efflux pathway external to the brain parenchyma, specifically, the perineural pathway along the optic nerves to evaluate if this pathway was compromised in GBM rats. Our data suggest that GBM rats have an impaired glymphatic system pathway within the brain parenchyma well as compromised perineural pathway along optic nerves. The glymphatic response to GBM may impact tumor cell migration, infiltration, and drug delivery administration via the ICM pathway.

## Materials and methods

### Animal tumor model and experimental methods

#### Rats

GBM-induced rats (3 months, male, Charles River’s immunodeficient Rowett Nude (RNU), *n* = 6) and age-matched control rats without tumor implantation (male, *n* = 8, Charles River’s Wistar, Wilmington, MA, US) were subjected to the same experimental procedures, including the ICM surgery for infusion of contrast agent and MRI measurements.

#### Cell culture

Primary human glioblastoma cells HF2354 were isolated from resected GBM tissue at Henry Ford Hospital and maintained in DMEM/F-12 medium (11,330,032, Thermo Fisher Scientific, MA) containing 25 µg/ml Gentamicin (G1272, Sigma-Aldrich, MA), Pen/Strep (1x, 15640-055, Gibco, MA), 1×N2 supplement (17,502,048, ThermoFisher Scientific, MA), 50 µg/ml BSA (A4919-5G, Sigma-Aldrich, MA), 20 ng/ml EFG (AF-100-15, Peprotech, NJ) and 20 ng/ml FGFb (100-18B, Peprotech, NJ).

#### Intracranial tumor Implantation

Nude rats were anesthetized with ketamine (80 mg/kg) and xylazine (13 mg/kg) administered intraperitoneally (IP). After being secured in a stereotaxic device, a 3–4 mm incision was made directly down the midline, the scalp was retracted, and the cranium was exposed. Using a drill, a 2 mm craniotomy was made on the right hemisphere anterior to the coronal suture. Tumor cells were injected intra-parenchymally into the right hemisphere using a 10 µl Hamilton syringe at 2.5 mm depth, 1.5 mm to the right, and 1.0 mm anterior to the bregma. A volume of 5 µl of HF2354 glioblastoma cells (5 × 10^5^) was injected intracerebrally. The craniotomy was sealed with bone wax and the incision was closed with a 4 − 0 silk suture.

#### Catheter Implantation into the cisterna magna

Prior to the MRI investigations, all rats were surgically prepped for catheter implantation into the cisterna magna for infusion. Isoflurane (3.0%) and a gas combination of N_2_O (70%) and O_2_ (30%) were used to anesthetize the rats, and once the rats were stable, the isoflurane was maintained in the range of 1.0 − 1.5%. After fixing the head in a stereotactic frame, the dorsal skin and muscle at the midline of the neck were incised, exposing the occipital bone. A 1 mm diameter hole was drilled through the skull using a Micromotor (Foredom Electric Co., Bethel, CT, USA), lateral to the midline of the skull and about 1 mm above the cisterna magna to expose the dura mater. A 27-gauge needle was used to puncture the dura mater. A part (approximately 2 mm long segment) of the polyethylene catheter (PE-10 tubing; Becton Dickinson, MD, US), approximately 10 µL in a volume filled with saline, was inserted into the cisterna magna. After that, the dura mater opening was sealed with glue, and a section of outer tubing was superglued to the occipital bone. Finally, the skin and muscle incisions were sutured. In order to prepare for infusion, Gd-DTPA (21.7mM) was loaded into a PE-10 catheter tube and linked to the indwelling catheter as an extension out of the MRI machine.

### MRI measurements

A 7 Tesla equipment (Bruker–Biospin, Billerica, MA, US) was used to acquire MRI measurements. A 2 × 2 surface array coil was utilized as the receiver, while a body volume birdcage-type coil was employed as the transmitter. The catheterized animal was securely fastened to an MR-compatible holder outfitted with an adjustable nose cone for anesthetic gas delivery and stereotaxic ear bars to restrain the head movement. The holder was pushed into the magnet and positioned at the center at the beginning of the MRI scan. A fast gradient echo imaging sequence was employed to assure the accurate positioning of the animal in the magnet. Animals’ respiration (50–65 breaths/minute) was monitored (Biopac Systems Inc., Goleta, CA, USA), and their anesthesia was maintained throughout MRI measurements using Isoflurane (1.0–1.5%) and a gas combination of N_2_O (70%) and O_2_ (30%) (Piramal Inc., Bethlehem, PA, US). An air heating blower (Rapid Electric, Brewster, NY, US) with feedback control was employed to keep the animals’ rectal temperatures at 36 ± 1 °C.

**Fig. 1:**
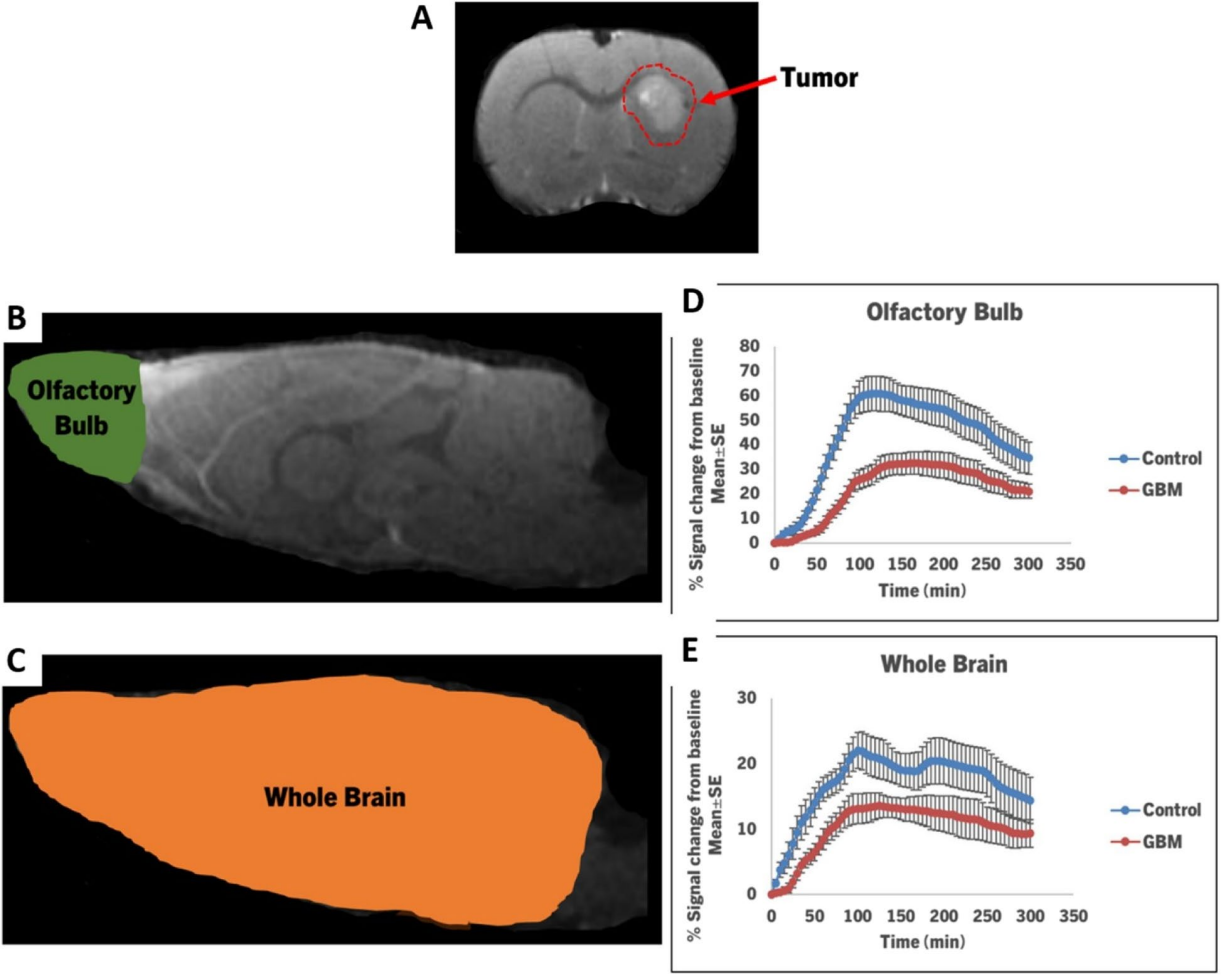
**A** T2WI shows the location of the tumor at 2.5 mm depth, 1.5 mm to the right, and 1.0 mm anterior to the bregma on the right hemisphere of the coronal section of the rat brain. The red dashed line encloses the whole tumor. Regions of interest (ROIs) for the olfactory bulb and whole brain are shown as colored anatomical areas in (**B, C**) on the sagittal sections of the rat brain. The time signal curves (TSCs) (**D, E**) are acquired from the related ROIs. Compared to the control rats, the TSCs measured from the GBM rats show an initial signal increase at later time points, a lower percentage of signal change from the baseline, and a slow signal decrease after the peak signal

To detect the tumor volume, coronal T2-weighted imaging (T2WI) with TE = 15, 30, 45, 60, 75, and 90 ms, TR = 3 s, FOV = 32 × 32 mm^2^, matrix = 256 × 256, thickness = 0.8 mm, slices = 15 was performed. The volume of the GBM was estimated as the sum of the tumor area in each slice multiplied by the slice thickness. Upon the attainment of the tumor volumes in the range of 22–77 mm^3^ (mean: 45.12 and standard deviation: 21.58) all rats were investigated using a dynamic CE-MRI technique as in previous investigations [5, 31]. Figure 1A shows the T2WI of the tumor enclosed in a red dashed line on the coronal section of the rat’s brain. To measure the dynamics of the CSF tracer through the glymphatic system, 3D T1WI (TE = 4 ms, TR = 18 ms, flip angle = 12º, FOV = 32 × 32 × 16 mm^3^, matrix = 256 × 192 × 96 (resolution of 0.125 × 0.167 × 0.167 mm) later interpolated to 256 × 256 × 96 voxels (resolution of 0.125 × 0.125 × 0.167 mm)) with paramagnetic contrast agent infusion of Gd-DTPA was performed. The 3D T1WI sequences continued for 5 h, beginning with three baseline scans prior to infusion, followed by ICM administration of Gd-DTPA (85 µl, 21mM) as a CSF tracer at an infusion rate of 1.67 µl/min for 50 min, using the indwelling catheter connected to a 100 µl glass syringe (Hamilton Robotics, Reno, NV, US) mounted on an infusion pump (Harvard Apparatus, Holliston, MA, US).

### Data analysis

Imaging was performed for up to 5 h after ICM infusion to track the CSF tracer distribution in the brain over time. Motion correction for the brain was accomplished using algorithms in MATLAB and by co-registering all the volumes to the first-time point volume [44]. Furthermore, the average of all co-registered and brain-extracted T1WIs for each animal was aligned to the reference animal to create a common spatial space for all the animals. The intensity value at each time, I_x_(t), was used to figure out the time signal curve (TSC), which shows how the density of the tracer changes over time in each voxel x.

1 $${TSC}_{x}\left(t\right)=\frac{{I}_{x}\left(t\right)-{I}_{x}\left({t}_{0}\right)}{{I}_{x}\left({t}_{0}\right)}$$

where t_0_ is the time before infusion.

Voxels of each brain were then clustered into comparable areas based on the tracer’s propagation profile during the experiment. Following clustering, the average TSC of the voxels within each cluster was allocated to that cluster. Benefiting from clustering the tissues, we identified a local input function for any formed cluster among the TSCs of its neighboring clusters. Compared to the previous modeling with the global input function obtained from the TSC of the whole brain [45], our local input function selected for each cluster reduces errors for the modeling of CSF tracer dynamics and provides a more accurate estimation of the parameters of the glymphatic system.

To better determine the kinetics of tracer influx and clearance following Gd-DTPA infusion, four parameters were extracted from the TSC for each tissue cluster. Arrival time (t_a_) is defined as the time at which the tracer enters each cluster location following infusion. Each cluster’s arrival time was calculated from its TSC by identifying the time at which the signal begins to increase and remains elevated for at least three subsequent time points. The infusion rate (IR) is defined as the slope of TSC between the time of arrival (t_a_) and the time at which TSC reaches its highest value (t_max_) throughout the accumulation phase for each cluster.

2 $$IR=\frac{TSC\left({t}_{max}\right)-TSC\left({t}_{a}\right)}{{t}_{max}-{t}_{a}}$$

The clearance rate is defined as the slope of TSC for each cluster between the time at which TSC achieves its highest value and the time at which TSC relaxes at the end of the experiment. Residual (Res) is the quantity of tracer that stays in the brain tissues at the end of an experiment.

3 $$Res\%=\frac{TSC\left({t}_{end}\right)-TSC\left({t}_{a}\right)}{TSC\left({t}_{max}\right)}\times 100$$

where TSC(t_a_) is equal to zero.

After calculating the kinetic parameters in each cluster from its average TSC, parametric scalar maps of these parameters were generated as previously described [46].

### Data quantification and statistical analysis

To evaluate the dynamics of CSF tracer distribution throughout the glymphatic system, regions of interest (ROIs) for the olfactory bulb and whole brain were manually identified on 3D T1WIs (Fig. 1B, C). TSCs were extracted from these particular areas using ROIs, and their kinetic characteristics were then evaluated between control and GBM rats. TSCs for each tissue ROI were normalized using the bone TSC for each animal. Evaluations of the parametric scalar maps and data quantifications were performed based on these ROIs. Results of each ROI for rats with and without GBM are shown as mean ± standard error (SE) (Fig. 1D, E). A two-sample t-test was conducted between two groups with a *p* < 0.05 statistical significance level in order to identify the effects of GBM on the glymphatic system.

## Results

### Signal intensity plots based on TSCs alterations with glioblastoma

TSCs acquired from the same brain areas (olfactory bulb and whole brain (Fig. 1B, C)) exhibited different temporal profiles between control and GBM rats as shown in Fig. 1D, E. TSCs from GBM rats revealed an initial signal rise at later time points, a reduced percentage of signal change from the baseline, and a delayed signal reduction after the peak signal as compared to the control rats. The slower rate of signal rise before peak values with less percentage of signal change from the baseline and a slower rate of signal drop after peak values seen in these brain areas in GBM rats imply a reduced influx and delayed clearance of CSF tracer via the glymphatic system, respectively.

### Impaired glymphatic influx and clearance of CSF tracer with glioblastoma

**Fig. 2:**
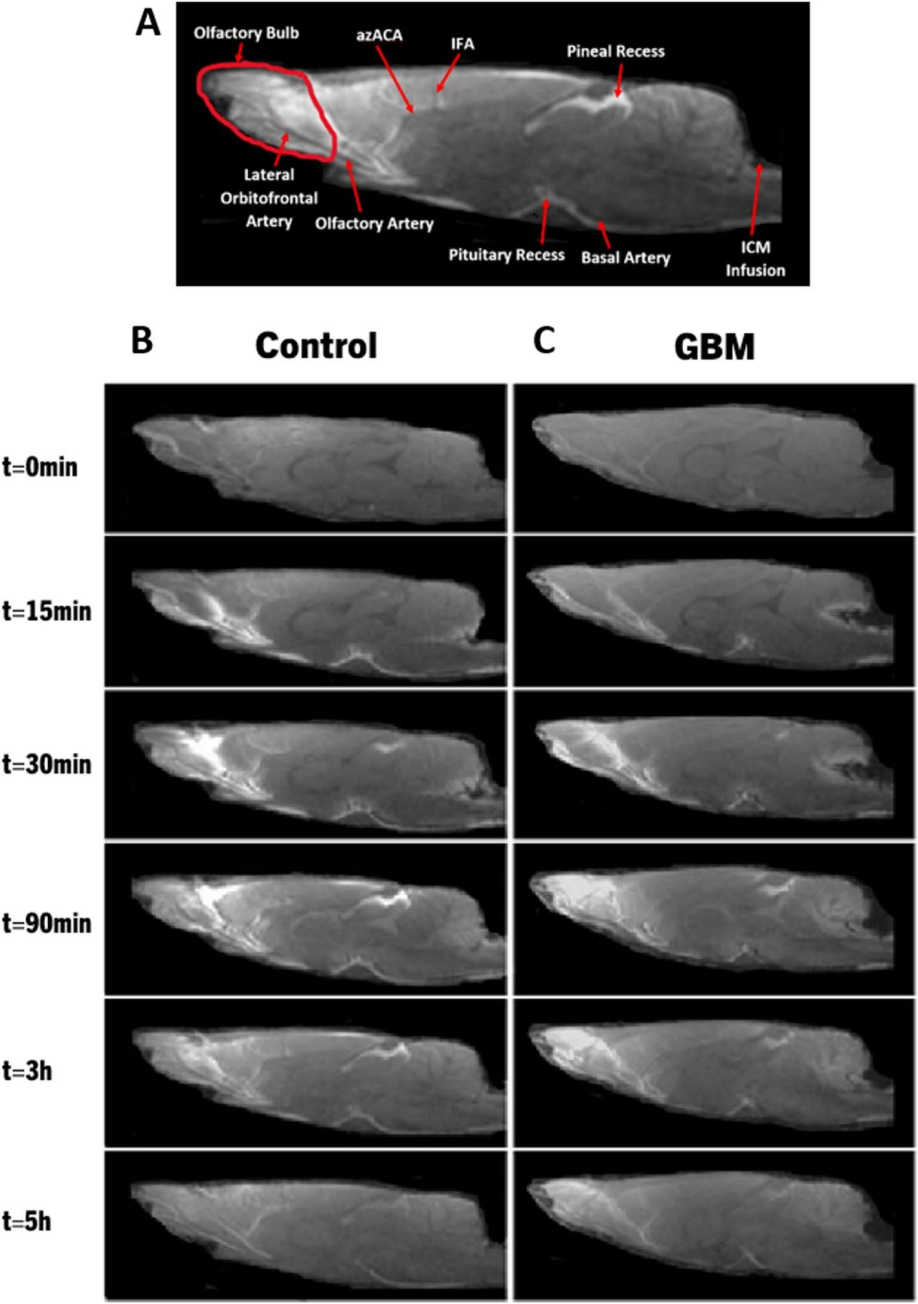
Dynamic tracer concentration changes in Control and GBM representative rats. **A **Visualization of key anatomical structures in the rat brain including the olfactory bulb, pituitary recess, pineal recess, ICM infusion site, and relevant arterial segments including the olfactory artery, lateral orbitofrontal artery, azygos of the anterior cerebral artery (azACA), and internal frontal artery (IFA). **B**, **C** shows the time evolution of contrast agent in control (**B**) and GBM (**C**) rats using CE-MRI, demonstrating influx (0-30 min) and anatomical glymphatic enhancement 90 min, 3 hours, and 5 hours after ICM infusion of Gd-DTPA

A comparison of CSF tracer transport through the glymphatic system over time in control and GBM rats is shown in Fig. 2. We investigated whether GBM has an impact on the glymphatic system influx and clearance mechanisms. During the 5-hour MR imaging session, the 3D dynamic T1WIs in representative control (Fig. 2B) and GBM (Fig. 2C) rats demonstrate the influx of Gd-DTPA contrast agent through time-dependent anatomical channels of perivascular spaces and other anatomical locations. The anatomical regions for the glymphatic system are marked in Fig. 2A. As shown in Fig. 2B, T1WIs in a control rat show the kinetics of Gd-DTPA as the CSF paramagnetic contrast agent enters the cisterna magna, the contrast agent enters the perivascular space of the basal artery, and the pituitary recess 15 min after infusion, the contrast agent movement along the olfactory artery, the olfactory bulb, and pineal recess 30 min after infusion, enhancement of contrast agent in the brain 90 min after infusion, clearance of contrast agent from the brain 3 h after infusion, and clearance of contrast agent from the brain at the end of the experiment (5 h after infusion). Within 15 min of infusion, the Gd-DTPA tracer was seen within the perivascular spaces around the arteries at the surface of the brain. Gd-DTPA entered the perivascular spaces of the penetrating arteries 30 min after infusion. Tracer entered the majority of brain regions and started to clear out 3 h after infusion, and most of the Gd-DTPA was cleared from the brain and olfactory bulb 5 h after infusion.

When compared to a control rat, the time-matched T1WIs in a GBM rat as shown in Fig. 2C show a different contrast agent distribution, including increased tracer intensity in the cerebellum (when a tracer is excessively intense, the signal saturates and appears as black area, as seen in the cerebellum of a GBM rat), decreased tracer intensity in the perivascular spaces around the arteries and the olfactory bulb, and increased tracer intensity at 5 h after Gd-DTPA infusion. Together, these data show that in GBM rats, more CSF tracer is retained in the cerebellum and less is taken up by the brain with less influx of contrast agent through the periarterial spaces, pituitary recess, and pineal recess than in control non-GBM animals. Additionally, GBM rats exhibit a decline in tracer clearance over time in the olfactory bulb and other brain regions. These dynamic images (Fig. 2) and their quantitative data (Fig. 1D, E) suggest the impaired glymphatic influx and clearance of CSF tracer in GBM rats.

### Reduced glymphatic flow of CSF tracer with glioblastoma

**Fig. 3:**
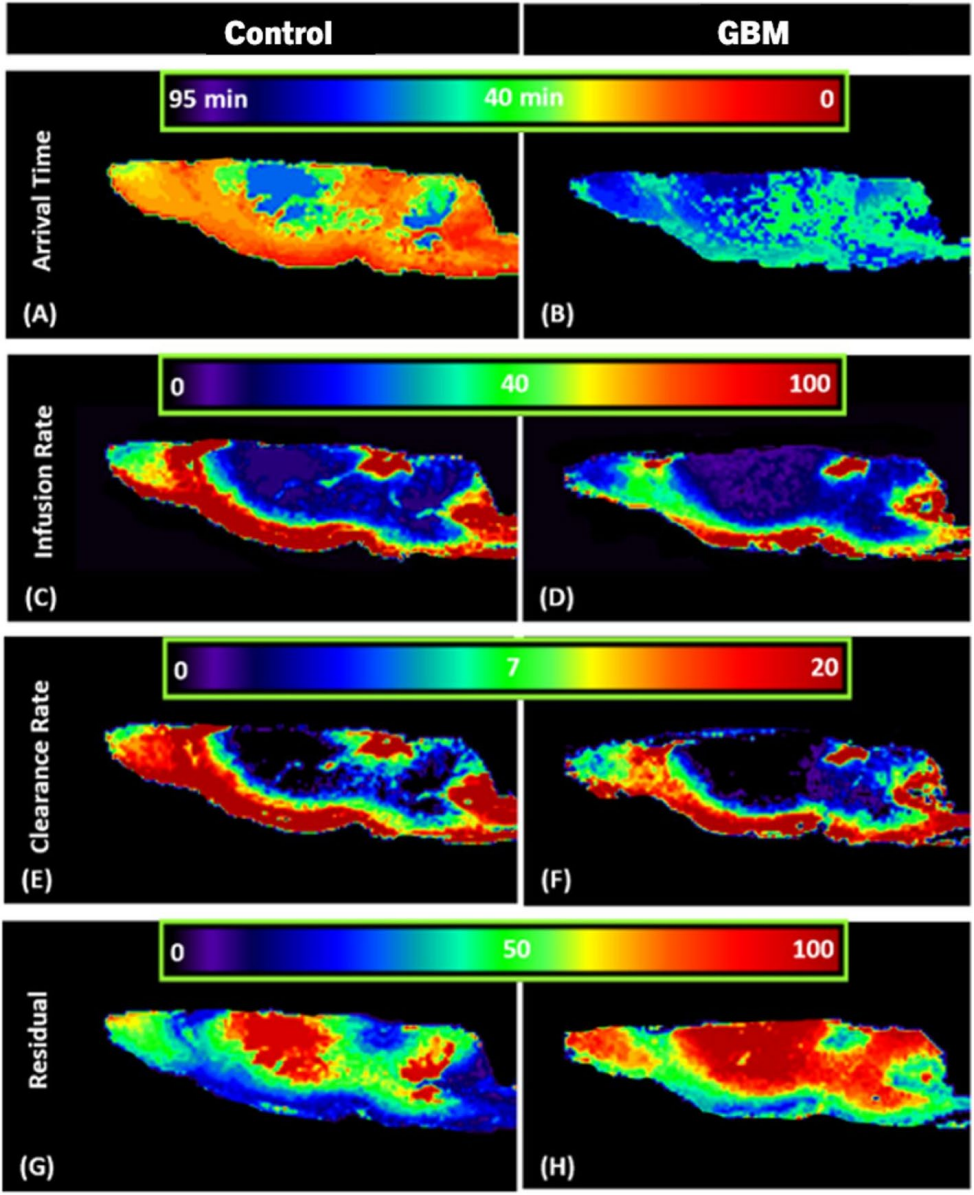
Scalar maps in Control and GBM representative rats. Hotter color (towards red) indicates quicker arrival time, faster infusion rate, faster clearance rate, and more residual of CSF tracer. **A**, **B** Arrival time maps suggest a lower bulk speed of CSF tracer in the periarterial spaces and olfactory bulb of GBM rats. **C**, **D** Infusion rate maps suggest a slower infusion of tracer in the periarterial spaces and olfactory bulb of GBM rats. **E**, **F** Clearance rate maps suggest slower clearance of tracer from the olfactory bulb and other brain regions of the GBM rats. **G**, **H** Residual maps suggest more tracer residual in GBM rats at the end of the experiment

Scalar maps of kinetic parameters were evaluated using our model of the glymphatic system [46] by calculating the TSCs of each cluster that represent the glymphatic flow of the CSF tracer in the brain. Figure 3 shows the scalar parametric maps of representative control and GBM rats. When the arrival time maps of control and GBM rats (Fig. 3A, B) were compared, it was observed that the tracer took longer to reach the perivascular spaces along the arteries in the GBM rat, reflecting a slower bulk speed of CSF in the perivascular spaces. Additionally, it took longer for the tracer to reach the olfactory bulb and the whole brain of the GBM rat as demonstrated in Fig. 3B. In comparison to the control rat (Fig. 3C), the infusion rate maps in Fig. 3D demonstrate delayed infusion in the perivascular spaces along the arteries in the GBM rat. Tracer infusion was also slower in the olfactory bulb and other brain regions of the GBM rat. Clearance rate maps indicate that the tracer was cleared more slowly from the olfactory bulb and whole brain of the GBM rat (Fig. 3F) than the control rat (Fig. 3E). Residual maps indicated that the GBM rat (Fig. 3H) retained more tracer than the control rat (Fig. 3G) throughout the brain and the olfactory bulb at the end of the experiment.

**Fig. 4:**
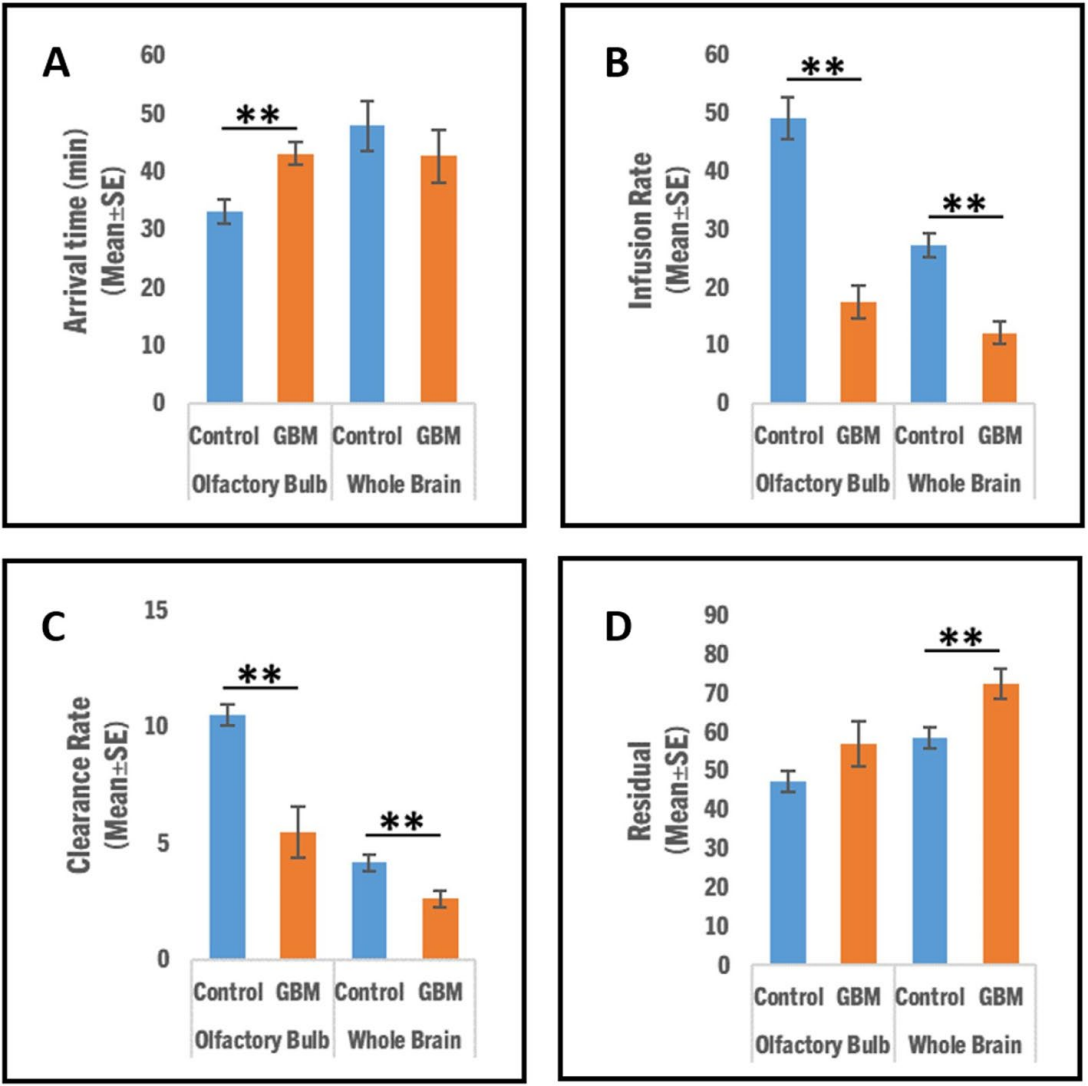
Comparison of glymphatic flow in the investigated brain areas (olfactory bulb and whole brain). Slower arrival time (**A**), significantly reduced infusion rate (**B**), significantly reduced clearance rate (**C**), and increased residual of tracer (**D**) in corresponding brain regions were found in the GBM rats compared to the control rats with * *p*<0.05, ** *p*<0.01

Figure 4 shows the quantitative comparison of arrival time, infusion rate, clearance rate, and residual of the CSF tracer in GBM and control group rats. Late arrival time of tracer (Fig. 4A; Olfactory Bulb: 43.15 ± 1.91 vs. 33.21 ± 2.10, *p* = 0.002; Whole Brain: 42.79 ± 4.59 vs. 47.95 ± 4.30, *p* = 0.21), significantly decreased infusion rate (Fig. 4B; Olfactory Bulb: 17.56 ± 2.88 vs. 49.23 ± 3.59, *p* < 0.001; Whole Brain: 12.23 ± 1.86 vs. 27.25 ± 2.01, *p* < 0.001), significantly decreased clearance rate (Fig. 4C; Olfactory Bulb: 5.51 ± 1.10 vs. 10.54 ± 0.45, *p* < 0.001; Whole Brain: 2.63 ± 0.36 vs. 4.17 ± 0.38, *p* < 0.001), and more tracer residual (Fig. 4D; Olfactory Bulb: 58.58 ± 2.80 vs. 47.24 ± 2.67, *p* = 0.06; Whole Brain: 72.70 ± 3.81 vs. 57.00 ± 5.76, *p* = 0.007) in the evaluated brain areas were found in the GBM rats compared to the control rats. Consistent with signal intensity plots (Fig. 1) and representative 3D T1WIs (Fig. 2), the scalar parametric maps (Fig. 3) and their quantified data (Fig. 4) with statistical differences between control and GBM rats suggest a compromised glymphatic flow in the GBM rats.

### Impaired perineural pathway along optic nerves with glioblastoma

**Fig. 5:**
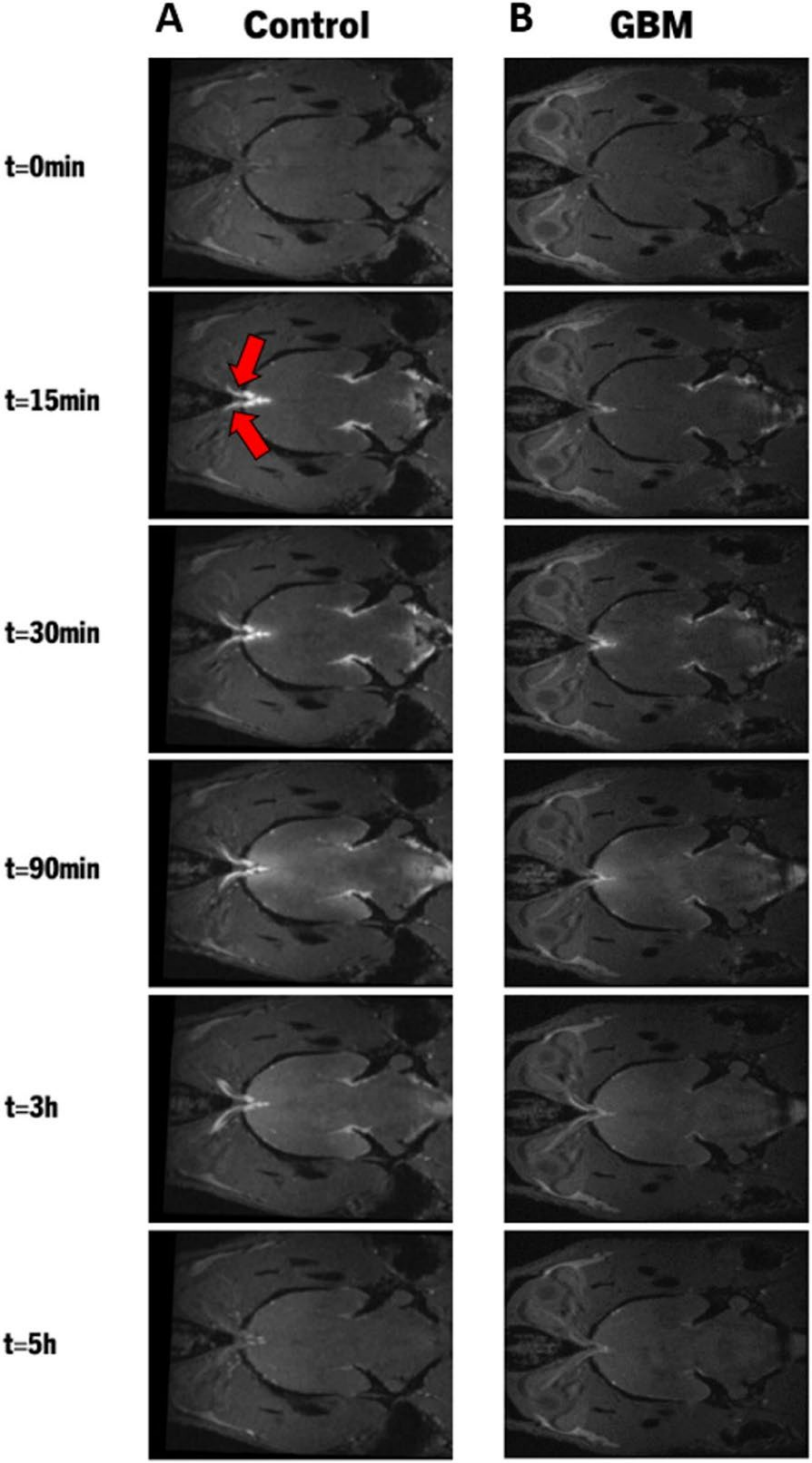
The perineural outflow pathway in Control and GBM rats. Red arrows point to the optic nerves. CE-MRI in representative Control (**A**) and GBM (**B**) rats show the dynamics of contrast agent in the perineural spaces along the optic nerves before infusion, 15 min, 30 min, 90 min, 3 hours, and 5 hours after the infusion of Gd-DTPA in the cisterna magna. These images suggest compromised outflow of CSF tracer from the perineural spaces along the optic nerves in GBM rat

In rodents, macromolecular tracers from the subarachnoid space are drained into the extracranial lymphatics through perineural pathways surrounding the departing cranial nerves, including the optic nerves [11, 47, 48]. We assessed the tracer intensity along the optic nerves in control and GBM rats to see if these perineural outflow channels were active in GBM rats. Figure 5 shows T1WIs of Gd-DTPA contrast along the optic nerves over time after ICM infusion. As shown in T1WIs, a representative GBM rat (Fig. 5B) exhibited less tracer along the perineural spaces of the optic nerves than a control rat (Fig. 5 A), indicating that this outflow pathway is compromised in GBM rats.

## Discussion

GBM is an aggressive type of brain tumor that may have a substantial influence on the glymphatic system, which is responsible for eliminating toxic interstitial waste solutes from the brain parenchyma. The typical path of the CSF through the brain may be compromised by GBM as these tumors grow with an increasing volume within brain tissue devoid of lymphatic vessels.

In this study, we investigated the response of the glymphatic system in rats with GBM using dynamic CE-MRI and advanced kinetic modeling. To the best of our knowledge, this is the first study to assess the glymphatic response to human-derived GBM in a rat model. The MRI data indicate that GBM rats have decreased glymphatic transport in the brain, with evidence of impaired glymphatic influx and clearance in the presence of GBM. Consistent with T1WIs, our parametric scalar maps, and representative quantified data identified a decreased glymphatic flow of CSF tracer in the GBM rats compared to the control rats. Our data also revealed a compromised CSF tracer efflux via the perineural spaces along the optic nerves in GBM rats.

The olfactory bulb is sensitive to changes in the glymphatic system [5, 43]. In order to evaluate the glymphatic system function in rats with GBM, we designated the olfactory bulb and whole brain as ROIs. The signal intensity plots evaluated using TSCs (Fig. 1D, E) showed a slower rate of signal increase before peak values with less percentage of signal change from the baseline and a slower rate of signal decline after peak values in GBM rats than in control rats, implying a reduced influx and delayed clearance of CSF tracer via the glymphatic system. Consistently, the dynamic tracer concentration changes in T1WIs over time in GBM rats indicated reduced perivascular CSF flow along basal, olfactory, and other arteries (Fig. 2C), as well as increased tracer retention at the end of the experiment, indicating an impaired glymphatic influx and clearance in GBM rats. The glymphatic system’s performance may be affected by tumor-associated inflammation, angiogenesis, and edema. Also, tumor growth may increase intracranial pressure (ICP), obstructing CSF flow and impairing the glymphatic system. AQP4 water channels are responsible for the periarterial influx of subarachnoid CSF into the brain parenchyma, the CSF-ISF exchange, and the perivenous efflux of CSF-ISF and interstitial waste solutes [5, 7]. Reduced glymphatic influx and clearance suggest alteration of astrocyte function and downregulation of AQP4 expression due to GBM. Reduced AQP4 expression with glioma has been demonstrated in a recent study [49].

Our study demonstrated the presence of Gd-DTPA tracer in the perivascular spaces along arteries and other anatomical routes that participate in the glymphatic system. However, reduced amounts of tracer entered into the brain of the GBM rats compared to the control rats, and more tracer was retained in the cerebellum (Fig. 2C), possibly due to elevated ICP and edema present in GBM rats than in control rats. Elevated ICP has been demonstrated in glioma rats [49]. This outcome is consistent with a recent study in glioma mice which demonstrated that after ICM infusion, less tracer entered the brain and was instead directed into the spinal space. They also demonstrated significant lymphatic outflow of CSF tracer from the sacral region in glioma versus control mice [50]. With cerebral edema and elevated ICP, CSF production also decreases in the brain [51, 52]. Reduced production and decreased outflow of CSF may have a significant impact on the glymphatic system, consistent with reduced glymphatic influx and clearance in the GBM rats observed in our study. In addition, the tumor and its microenvironment may hinder the glymphatic pathway which affects the influx and clearance of CSF tracer.

We employed advanced kinetic modeling to seek a better understanding of the function of the glymphatic system with GBM, and our efforts focused on the parametric scalar maps (Fig. 3) and quantitative analysis (Fig. 4). Scalar maps suggested slower arrival of CSF tracer in the GBM rats (Figs. 3B and 4A) than in the control rats, suggesting a reduced bulk speed of CSF in the perivascular spaces as compared to the control rats. Scalar maps also suggested significantly slower infusion of tracer (Figs. 3D and 4B), significantly slower clearance of tracer (Figs. 3F and 4C), and more tracer residual (Figs. 3H and 4D) in the GBM rats than in the control rats. The parametric scalar maps and regional quantifications with statistical differences between control and GBM groups suggest a compromised glymphatic flow in the GBM rats. Reduced glymphatic flow and clearance may cause toxic solutes and pro-inflammatory signaling molecules such as cytokines and chemokines to accumulate in the brain, causing persistent inflammation and thereby possibly encouraging the growth of GBM.

Our data also demonstrated reduced CSF tracer efflux through the perineural spaces of optic nerves in GBM rats (Fig. 5B), indicating compromised perineural pathway. These data are also consistent with a prior study demonstrating that glioma mice have impaired perineural outflow channels [50]. Our data show that the Gd-DTPA signal in the olfactory bulb, as well as in the entire brain of control rats, significantly decreased over time after reaching peak intensity; however, in contrast, the signal intensity was relatively steady in GBM rats (Fig. 1D, E). This difference between GBM and control rat data is indicative of a reduced glymphatic clearance in GBM rats to further drain to the extracranial lymphatic vessels. Our results are consistent with earlier work, which showed that glioma mice exhibited reduced CSF drainage through the extracranial lymphatics and had significantly reduced signal intensity in the deep cervical lymph nodes and mandibular lymph nodes compared with control mice [50]. Reduced CSF outflow would likely result in less tumor-specific antigen being drained to cervical lymph nodes, resulting in reduced anti-tumoral T-cell activation and a weaker immunological response.

Basal meningeal lymphatic vessels have been demonstrated as hotspots for CSF macromolecules drainage [37]. The dorsal meningeal lymphatic vessels, on the other hand, have been shown to provide a pathway for tumor cells to drain into deep cervical lymph nodes and potentially contribute to extracranial metastasis [36]. However, within the brain parenchyma, the tumor cells invade the brain tissue diffusely via active cell migration [53, 54] primarily driven by the attachment-detachment mechanism of glioma cells and the dynamic remodeling of extracellular matrix [42]. Tumor cells also invade other brain regions via the perivascular spaces and white-matter tracts [42]. While the precise association between the glymphatic system and GBM cell infiltration is unknown, the glymphatic system convective flux could play an important role in tumor cell migration. Future studies are required to determine the precise relationship between these processes. Understanding if and how the glymphatic system influences GBM growth may lead to the identification of new treatment strategies for inhibiting GBM cell invasion and increasing the survival rate of patients.

A contributing factor to the essentially ineffective treatment outcomes of GBM is that invading tumor cells are not exposed to the existing standard-of-care therapies. Generally, the therapeutic antibodies are administered by intravenous infusion [55, 56]. However, only a few antibodies enter the brain tissue due to the presence of the BBB [57]. While antibodies have restricted diffusive transport in the brain’s extracellular space [58, 59], glymphatic perivascular and intra-parenchymal convective flow may be used to improve their delivery into the brain. Increasing plasma osmolality by injecting hypertonic saline or mannitol intraperitoneally has been shown to lower the ICP while increasing the glymphatic influx of ICM-injected antibodies without disruption of the BBB [60]. This technique overcomes the impaired glymphatic influx seen in the brain of awake mice and has been shown to effectively improve the delivery of an Aβ antibody, achieving a 5-fold increment in antibody binding to Aβ plaques while using significantly fewer antibodies in an AD model [60]. Thus, utilizing the brain-wide system of perivascular spaces and enhancing the glymphatic activity by increasing plasma osmolality and lowering the ICP may augment the delivery of anti-tumor drugs to the patients. Future research on utilizing the glymphatic pathway as a means to enhance drug delivery in GBM patients warrants investigation.

Additional studies measuring the glymphatic system are required due to the heterogeneity of GBM and the impact that age, gender, tumor size, and stage of the disease may have on the CSF flow. The anatomical and physiological (such as brain mass, metabolic rate, vascular pulsatility, AQP4 density, etc.) distinctions between human and rodent brains should be kept in mind when translating animal experimental results to humans.

## Conclusions

To assess the response of the glymphatic system to GBM, we employed GBM-induced RNU rats and CE-MRI to dynamically monitor the Gd-DTPA contrast agent infused into the cisterna magna. 3D T1WI measurements indicate reduced tracer entry into the brain of GBM rats with some of the tracer confined in the cerebellum compared with control rats. Dynamic T1WIs and signal intensity plots reveal a delayed periarterial influx of CSF tracer and a reduced glymphatic clearance in GBM rats compared with control rats. Parametric scalar maps and regional quantitative analysis derived using kinetic modeling demonstrate significantly slower tracer arrival, slower periarterial glymphatic influx, delayed glymphatic clearance, and more retention of tracer in the GBM rats than in control rats. Our study also indicates the compromised CSF tracer efflux through the perineural spaces of the optic nerves with GBM. Consideration should be given to utilizing the brain-wide perivascular glymphatic pathway along with increasing plasma osmolality to increase drug delivery through ICM for the treatment of GBM.

## Data Availability

The datasets used and/or analyzed during the current study are available from the corresponding author upon reasonable request.

## Abbreviations

GBM: Glioblastoma Multiforme
CSF: Cerebrospinal fluid
ISF: Interstitial fluid
CE-MRI: Contrast-enhanced magnetic resonance imaging
T1WI: T1-weighted imaging
ICM: Intra-cisterna magna
AQP4: Aquaporin-4
BBB: Blood-brain-barrier
Aβ: Amyloid-beta
AD: Alzheimer’s disease
SVD: Small vessel disease
TBI: Traumatic brain injury
CLNs: Cervical lymph nodes
TSC: Time signal curve
ROIs: Regions of interest
ICP: Intracranial pressure
SE: Standard error
